# Multicore and GPU algorithms for Nussinov RNA folding

**DOI:** 10.1186/1471-2105-15-S8-S1

**Published:** 2014-07-14

**Authors:** Junjie Li, Sanjay Ranka, Sartaj Sahni

**Affiliations:** 1 Department of Computer and Information Science and Engineering, University of Florida, 32611 Gainesville, USA

**Keywords:** Nussinov, Multicore, CUDA, GPU, RNA

## Abstract

**Background:**

One segment of a RNA sequence might be paired with another segment of the same RNA sequence due to the force of hydrogen bonds. This two-dimensional structure is called the RNA sequence's secondary structure. Several algorithms have been proposed to predict an RNA sequence's secondary structure. These algorithms are referred to as RNA folding algorithms.

**Results:**

We develop cache efficient, multicore, and GPU algorithms for RNA folding using Nussinov's algorithm.

**Conclusions:**

Our cache efficient algorithm provides a speedup between 1.6 and 3.0 relative to a naive straightforward single core code. The multicore version of the cache efficient single core algorithm provides a speedup, relative to the naive single core algorithm, between 7.5 and 14.0 on a 6 core hyperthreaded CPU. Our GPU algorithm for the NVIDIA C2050 is up to 1582 times as fast as the naive single core algorithm and between 5.1 and 11.2 times as fast as the fastest previously known GPU algorithm for Nussinov RNA folding.

## Background

An RNA sequence is a chain of nucleotides from the alphabet {*G *(guanine), *A *(adenine), *U *(uracil), *C *(cytosine)}. One segment of a RNA sequence might be paired with another segment of the same RNA sequence due to the force of hydrogen bonds. This two-dimensional structure is called the RNA sequence's secondary structure. Two nucleotides in an RNA sequence can form Watson-Crick *AU *and *GC *base pairs as well as the *GU *wobble pair. Several algorithms have been proposed to predict an RNA sequence's secondary structure. These algorithms are referred to as RNA folding algorithms. Waterman and Smith [1] and Nussinov et al. [2] made the first attempt in 1978. Zuker et al. [3] refined Nussinov's algorithm by using a thermodynamic energy minimization model, which produces more accurate results at the expense of greater computational complexity. Although our focus in this paper is the simpler Nussinov's algorithm, our strategies may be applied to Zuker's algorithm as well.

The complexity of Nussinov's and Zuker's algorithm is *O*(*n*^3^), where *n *is the length of the RNA sequence to be folded. Other RNA folding algorithms with better prediction properties and higher complexity also exist. When folding viral sequences, *n *ranges from several thousand to several million and single-core run time becomes excessive and so much effort has gone into developing parallel versions of various RNA folding algorithms. For example, [4,5] develop a multicore code for an *O*(*n*^4^) folding algorithm while [6] does this for Nussinov's algorithm. [7] develops a framework for RNA folding algorithms on a cluster and tests this framework using an *O*(*n*^6^) (Pknots-RE) and an *O*(*n*^4^) (Pknots-RG) algorithms for the prediction of RNA secondary structure. FPGA solutions for secondary structure prediction have been proposed in [8-10] and GPU solutions in [11,12]. We note that [11] is based on the algorithm of Zuker et al. [3] while [12] is based on that of Nussinov et al. [2].

We start in this paper by describing the GPU architecture and programming model. Then we state Nussinov et al.'s [2] dynamic programming recurrence for secondary structure prediction and we give modifications of these equations as obtained by Chang et al. [12]. These modifications simplify the parallelization of the original equations and compute the same results. We also describe the strategy used in [12] to obtain a GPU algorithm to solve the modified equations. A naive implementation of the modified equations of [12] together with a cache efficient version and multicore versions are given. We note that although [6] gives a multicore version of Nussinov's algorithm, our multicore version is much simpler and provides similar speedup. Then our GPU algorithm for the modified equations is described. Experimental and benchmark results are presented after that.

### GPU architecture and programming model

**Figure 1:**
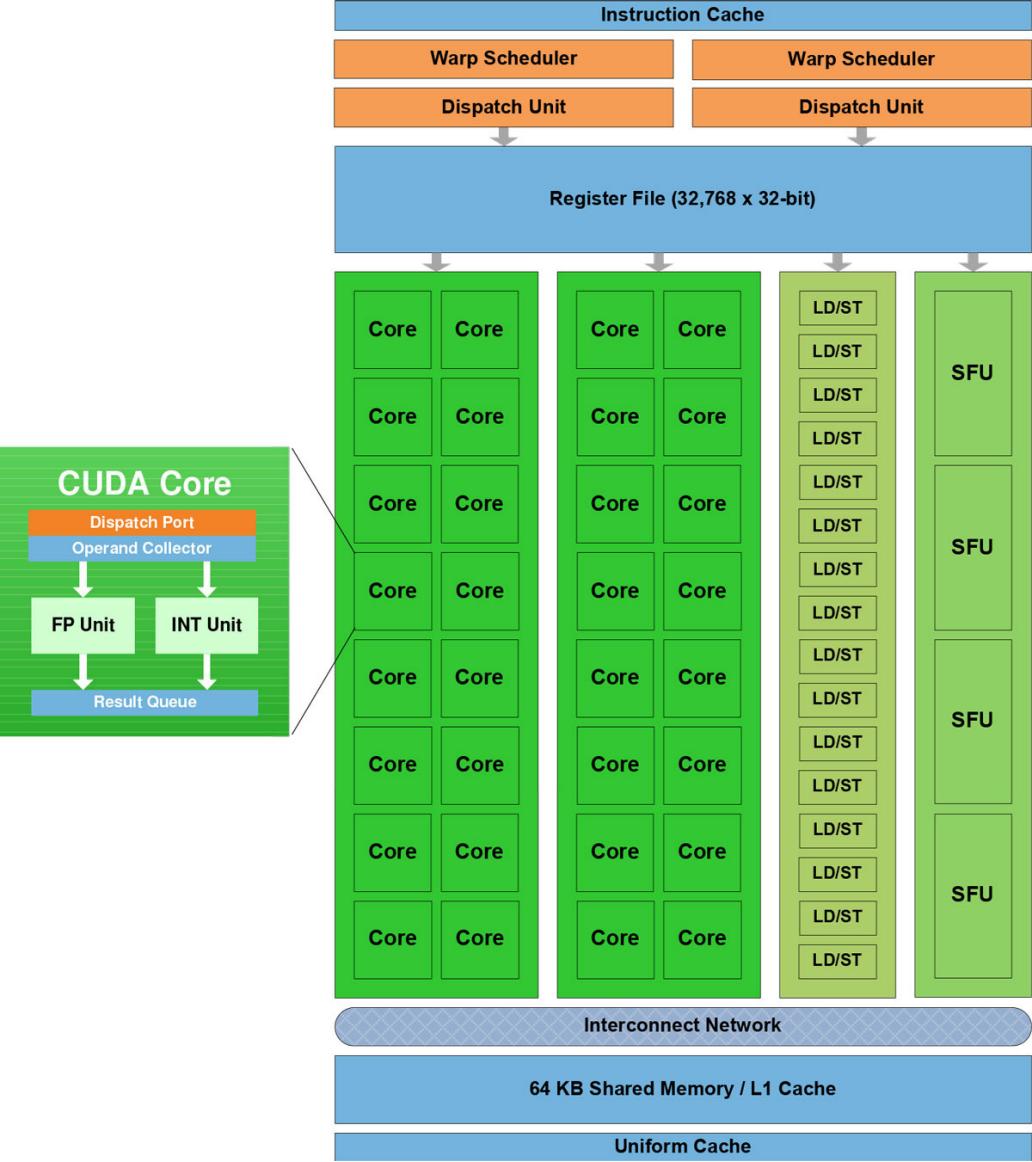
**Architecture of one SM of the NVIDIA Fermi **[14].

Our work targets the NVIDIA C2050 GPU. Figure 1 shows the architecture of one streaming multiprocessor (SM) of the NVIDIA Fermi line of GPUs of which the C2050 is a member. The C2050 comprises 448 processor cores grouped into 14 SMs with 32 cores per SM. Each SM has 64KB of shared memory/L1 cache that may be set up as either 48KB of shared memory and 16KB of L1 cache or 16KB of shared memory and 48KB of L1 cache. In addition, each SM has 32K registers. The 14 SMs access a common 3GB of DRAM memory, called device or global memory, via a 768KB L2 cache. A C2050 is capable of performing up to 1.288 TFLOPS of single-precision operations and 515 GFLOPS of double precision operations. A C2050 connects to the host processor via a PCI-Express bus. The master-slave programming model in which one writes a program for the host or master computer and this program invokes kernels that execute on the GPU is supported. GPUs use the SIMT (single instruction multiple thread) programming model in which the GPU accomplishes a computational task using thousands of light weight threads. The threads are grouped into blocks and the blocks are organized as a grid. While a block of threads may be 1-, 2-, or 3-dimensional, the grid of blocks may only be 1 or 2-dimensional. The key challenge in deriving high performance on this machine is to be able to effectively minimize the memory traffic between the SMs and the global memory of the GPU. This effectively requires design of novel algorithmic and implementation approaches and is the main focus of this paper.

### Nussinov's dynamic programing recurrence

Let *S *= *a*_1_*a*_2_...*a_n _*be an RNA sequence where *a_i _*ϵ {*A, C, G, U*} is a nucleotide. Nussinov's algorithm finds the most possible secondary structure by maximizing the number of bonded pairs (AU, GC or GU). Let *C*(*i*, *j*) be the maximum number of bonded pairs in the subsequence *a_i _*⋯ *a_j_*, 1 ≤ *i *≤ *j *≤ *n*. Nussinov's dynamic programming recurrence for *C *is given below.

$$\begin{array}{ccccc} C\left(i,\;i-1\right) & = & 0 & & 2\leq i\leq n\\ C\left(i,\;i\right) & = & 0 & & 1\leq i\leq n \end{array}$$

$$C\left(i,j\right)=\text{max}\left\{\begin{array}{c} C\left(i+1,j\right)\\ C\left(i,j-1\right)\\ C\left(i+1,j-1\right)+bond\left(a_{i},\;a_{j}\right)\\ {\text{max}}_{i<k<j}\left\{C\left(i,\;k\right)+C\left(k+1,\;j\right)\right\} \end{array}\right)$$

Here, bond(*a_i_, a_j _*) is 1 if (*a_i_, a_j _*) is an AU, GC or GU pair and 0 otherwise, and the third equation applies when *i *<*j*. The third equation computes the maximum of four terms that have the following significance.

**Figure 2:**
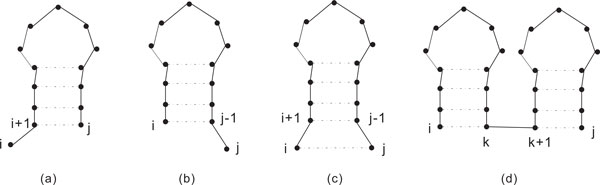
**Four cases in Nussinov's recurrence **[15].

1 Add unpaired *a_i _*to the best structure for subsequence *a_i_*_+1_...*a_j _*, as shown in Figure 2(a).

2 Add unpaired *a_j _*to the best structure for subsequence *a_i_*...*a_j_*_−1_, as shown in Figure 2(b).

3 Add (*a_i_, a_j _*) pair to the best structure for subsequence *a_i_*_+1_...*a_j_*_−1_, as shown in Figure 2(c).

4 Combine two best structures for *a_i_*...*a_k _*and *a_k_*_+1_...*a_j_*, as shown in Figure 2(d).

Once the *C*(*i, j*), 1 ≤ *i *<*j *≤ *n*, have been computed, a traceback procedure can be used to construct the actual secondary structure, which is represented in the matrix as a path leading to the maximum score. While this traceback procedure takes *O*(*n*) time, the actual computation of the *C *matrix takes *O*(*n*^3^) time.

### Simplified recurrence and GPU algorithm

Chang et al. [12] simplified Nussinov's recurrence to the following.

(1) C(i,i)=0for1≤i≤n

(2) C(i,i+1)=0for1≤i≤n-1

(3) $$C\left(i,j\right)=\text{max}\left\{\begin{array}{c} C\left(i+1,j-1\right)+bond\left(a_{i},a_{j}\right)\\ {\text{max}}_{i\leq k<j}\left\{C\left(i,k\right)+C\left(j,k+1\right)\right\} \end{array}\right)$$

**Figure 3:**
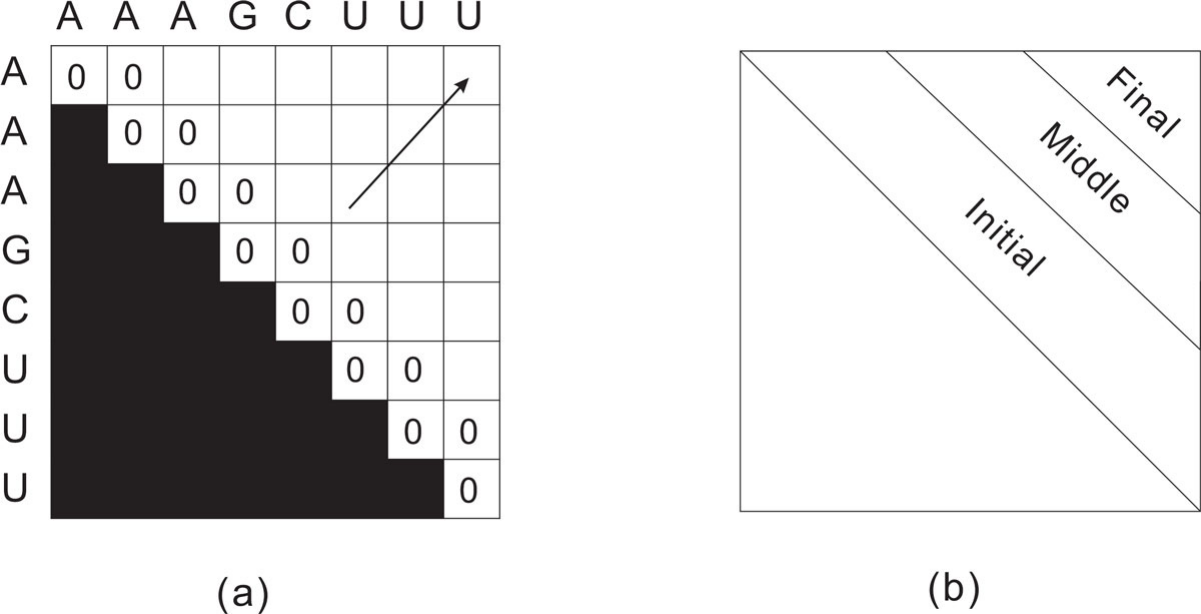
**(a) Initialization of the matrix in Chang's algorithm **[12]. (b) Three stages in Chang's algorithm.

Like Nussinov's original recurrence, the simplified recurrence uses *O*(*n*^2^) memory and *O*(*n*^3^) time. However, Chang's formulation is easier to parallelize. In a serial computation of *C*, we would start by initializing *C*(*i, i*) (the main diagonal of *C*) and *C*(*i, i *+ 1) (the diagonal just above the main one) using Equations 1 and 2 and then use Equation 3 to compute the remaining diagonals in the order *C*(*i, i *+ 2) ... *C*(*i, i *+ *n *− 1). Figure 3(a) shows how the computation progresses.

In [12], the entire computation is divided into three stages as shown in Figure 3(b), namely the initial stage, the middle stage and the final stage. In the initial stage, since the computation at each element is shallow (the distance to the central diagonal is short), one GPU thread is assigned to compute one element. No data exchange between threads is needed. All threads synchronize before moving to the next diagonal. In the middle stage, an entire block of threads is assigned to compute one element and the parallel reduction method contained in CUDA SDK is used. In the final stage, all SMs collaborate to compute one element because the distance from the element to the central diagonal is long and the computation for each element is heavy. Again, parallel reduction is used in this stage. To reduce accesses to device memory, the GPU algorithm of [12] stores each *C*(*i, j*) value, *i *<*j *in positions (*i, j*) as well as in the otherwise unused position (*j, i*). When *C*(*j, k *+ 1) is to be read from device memory (i.e., when it is needed in the right side of Equation 3), the read is done from position (*k *+ 1, *j*). This changes column reads to row reads and better utilizes the L2 and L1 caches of the target GPU.

## Methods

### Cache efficient and multicore algorithms

*CPU*1 (Algorithm 1) is a naive single-core algorithm to compute *C *using the simplified recurrence of Chang et al. This algorithm computes *M *[*i*][*j*] = *C*(*i*+1, *j*+1), 0 ≤ *i *≤ *j *<*n*, where *n *is the length of the RNA sequence *R*.

**Algorithm 1 ***CPU*1

**Require: **RNA sequence *R*

**Ensure: **Array *M *such that *M *[*i*][*j*] = *C*(*i *+ 1, *j *+ 1)

1: **for ***i *← 0 to |*R*|-1 **do**

2:    *M *[*i*][*i*] ← 0

3: **end for**

4: **for ***i *← 0 to |*R*|-2 **do**

5:    *M *[*i*][*i *+ 1] ← 0

6: **end for**

7: **for ***diag *← 2 to |*R*|-1 **do**

8:    **for ***row *← 0 to |*R*|-*diag*-1 **do**

9:        *col *← *diag *+ *row*

10:        *a *← *R*[*row*]; *b *← *R*[*col*]

11:        *max *← *M *[*row *+ 1][*col *− 1] + *bond*(*a, b*)

12:        **for ***k *← *row *to *col*-1 **do**

13:            *t *← *M *[*row*][*k*] + *M *[*k *+ 1][*col*]

14:            *max *← *max*{*max, t*}

15:        **end for**

16:        *M *[*row*][*col*] ← *max*

17:    **end for**

18: **end for**

By using the lower triangle of *M *to store the transpose of the computed values in the upper triangle of *M *as is done in the GPU implementation of [12], we get a cache efficient version of *CPU*1. To arrive at *CPU*2, we change Line 13 of Algorithm 1 to "*t *← *M *[*row*][*k*] + *M *[*col*][*k *+ 1]", and change Line 16 to "*M *[*row*][*col*] ← *M *[*col*][*row*] ← *max*". Values of *M *[*k *+ 1][*col*] locate in the same column but values of *M *[*col*][*k *+ 1] locate in the same row, for row ≤ *k *< col. Reading values in a row is more cache efficient than reading values in a column.

Multicore versions of *CPU*1 and *CPU*2, respectively labeled *OMP*1 and *OMP*2, are obtained by inserting OpenMP statements to parallelize the for loops of lines 1, 4, and 8.

### Our GPU algorithm

**Figure 4:**
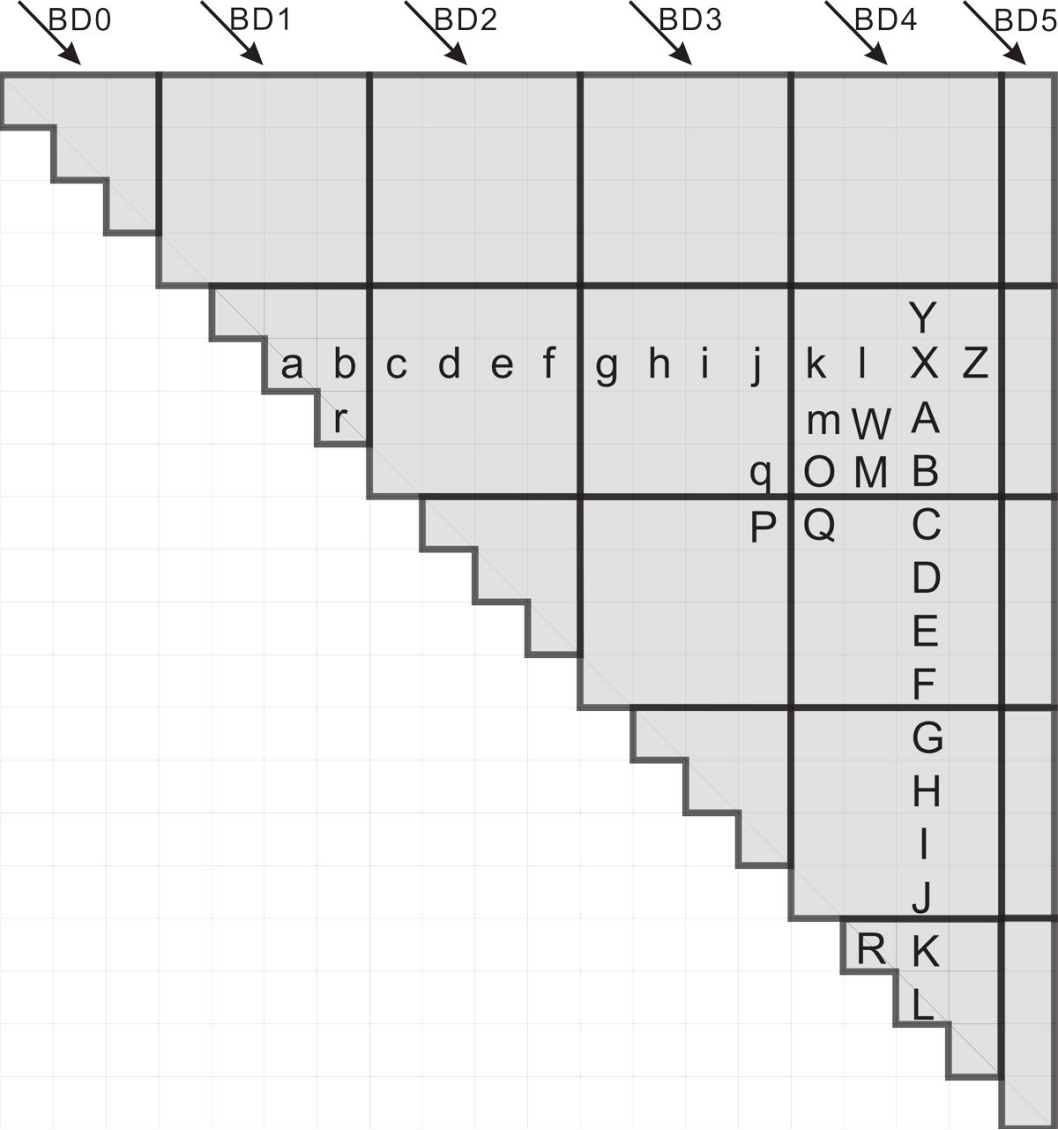
**Block partitioning of C matrix**.

Unlike the GPU algorithm of [12] which computes *C *by diagonals, we use a refinement of the block strategy used in [11,6]. Figure 4 shows the 20 × 20 C matrix for the case of RNA sequences whose length is *n *= 20. To compute the element labeled "X", elements "a" to "l" are, respectively, added to elements "A" to "L" and the maximum of "a+A", "b+B", ... "l+L" is computed. We note that the computation for "Y" also requires "A" to "L" and that "X" has to be computed before "Y" and "Z" can be computed.

In our block strategy, we partition the upper triangle of the *C *matrix into square blocks except that adjacent to the main diagonal the partitioning is into triangles and that at the right end is into possibly non-square rectangles. Figure 4 shows the partitioning for the case *n *= 20 using 4 × 4 blocks. Notice the triangles adjacent to the main diagonal and the 4 × 1 non-square partitions at the right end. The blocks (whether triangular, square, or non-square) are indexed left-to-right top-to-bottom beginning with (0, 0). In keeping with the traditional way to number blocks for GPU computation, the first coordinate increases as you move to the right and the second as you move down. So "X" (Figure 4) resides in (4, 1), "K" in (4, 4), and "P" in (3, 2). Blocks on the main diagonal are indexed (*i, i*) and are triangular. For the dependencies in Equation 3, it follows that blocks that lie on the same diagonal of blocks (i.e., blocks with the index (*i, i *− *k*) for some fixed *k*) are independent and so may be computed in parallel but that elements within a block are to be computed by diagonals. Our 20 × 20 example of Figure 4 has 6 diagonals of blocks and so six iterations of computation with each iteration computing all blocks on the same diagonal in parallel are required.

As noted earlier, the first diagonal of blocks is comprised of triangles. To each triangular block, we assign a thread block. The threads within the assigned thread block compute the elements in the triangular block in diagonal order with all elements on the same diagonal being computable in parallel. Hence, for this computation, the number of thread blocks equals the number of triangular blocks.

Let us turn our attention now to the remaining blocks (i.e., the non-triangular blocks). Notice that when we start the computation of the elements in, say, block (4, 1), where "X" resides, "a" to "j", and "C" to "L" have already been computed, because they are on preceding block diagonals. But "k", "l", "A", and "B" have yet to be computed. The computation of the maximum of "c+C" to "j+J" can be done using a kernel *maxKernel *(described later). This kernel uses registers for temporary values and writes these temporary values to shared memory upon completion. The final value for "O" can be obtained by comparing the temporary maximum value in "O" with "P" plus the *bond *value in Equation 3. Then the maximum of "r+O", "q" plus its *bond *value, and the temporary maximum value in "m" is written to "m" as its final value. Similarly, for "M", the maximum of "O+R", "Q" plus its bond value, and the temporary maximum value in "M" is written to "M" as its final value. The computations for "m" and "M" can be done in parallel. So the computation within element block (4, 1) is done in diagonal order. All elements on the same diagonal can be computed in parallel with all data residing in shared memory. The pseudocode is shown as Algorithm 2.

**Algorithm 2 **Our GPU algorithm

**Require: **RNA sequence *R*, blocked diagonal index *D*, block size *BS*

**Ensure: ***C *matrix

1: *Register*[16] *reg*

2: *Shared*_*Memory*[*BS*][*BS*] *sA*

3: *Shared_Memory*[*BS *+ 1][*BS *+ 1] *sC*

4: *Global*_*Memory*[*BS*][*BS*] *gB*

5: *Global*_*Memory*[*BS*][*BS*] *gC*

6: *tx *← *threadIdx.x*; *ty *← *threadIdx.y*

7: *bx *← *blockIdx.x*; *by *← *blockIdx.y*

8: *aRow *← *by *× *BS*; *aCol *← *aRow *− 1

9: *bRow *← *aRow*; *bCol *← *D *× *BS *− 1 + *aRow*

10: **for ***blk *← 1 to *D *− 1 **do**

11:        *sA *← the block starting at (*aRow, aCol *+ *blk *× *BS*)

12:        *gB *← the block starting at (*bRow *+ *blk *× *BS, bCol*)

13:        *maxKernel*(*sA, gB, reg*)

14:        *Syncthreads*

15: **end for**

16: *sC *← *reg*

17: **for ***d *← 1 to *BS *× 2 − 1 **do**

18:        **for **Each element *e *on diagonal *d ***do**

19:            Finish remaining computation

20:        **end for**

21:        *Syncthreads*

22: **end for**

23: *gC *← *sC*

**Algorithm 3 ***maxKernel*

**Require: **Block *sA *in shared memory, block *gB *in global memory

**Ensure: **Partial result of reduction in *reg*

        *r*0 ← *gB*[0][*tx*]; *r*1 ← *gB*[1][*tx*]

        *r*2 ← *gB*[2][*tx*]; *r*3 ← *gB*[3][*tx*]

        **for ***j *← 0 to 6 **do**

            **for ***k *← 0 to 15 **do**

                *reg*[*k*] ← max{*reg*[*k*], *r*0 + *sA*[*ty *× 16 + *k*][*j *× 4]}

            **end for**

            *r*0 ← *gB*[(*j *+ 1) × 4][*tx*]

            // 2 similar for loops for r1 and r2 come here

            for *k *← 0 to 15 **do**

                *reg*[*k*] ← max{*reg*[*k*], *r*3 + *sA*[*ty *× 16 + *k*][*j *× 4 + 3]}

            **end for**

            *r*3 ← *gB*[(*j *+ 1) × 4 + 3][*tx*]

        **end for**

        **for ***k *← 0 to 15 **do**

            *reg*[*k*] ← max{*reg*[*k*], *r*0 + *sA*[*ty *× 16 + *k*][28]}

        **end for**

        // 2 similar for loops for r1 and r2 come here

        **for **k ← 0 to 15 **do**

            *reg*[*k*] ← max{*reg*[*k*], *r*3 + *sA*[*ty *× 16 + *k*][31]}

        **end for**

#### Description of maxKernel

**Figure 5:**
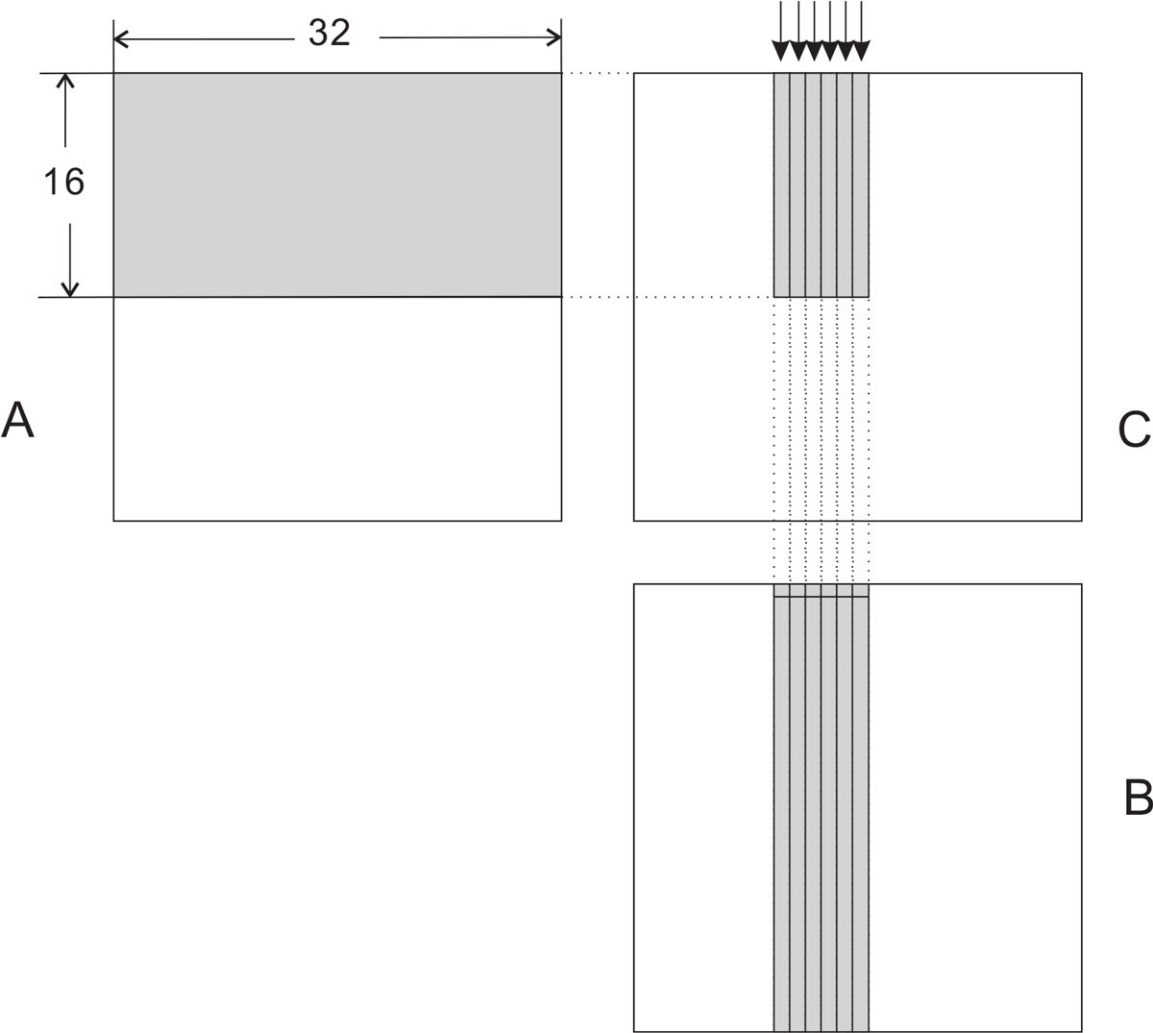
***maxKernel *illustration**.

The computation of the maximum of "c+C" to "j+J" (Figure 4) bears some resemblance to the computation of a term in a matrix multiply. So, we can adapt the ideas used in matrix multiply kernels to arrive at an efficient kernel to find the desired maximum of the sum of pairs. In our case (Algorithm 3), we adapt the GPU matrix multiply kernel of Volkov and Demmel [13]. The element block size used in our implementation is 32 × 32 and a thread block is configured as 32 × 2. Each thread computes 16 elements that lie in the same column as shown in Figure 5 (this figure shows only six threads as arrows above block C). The 16 elements computed by one thread are represented as a slim gray bar in block *C*. The gray area in block A depicts the data needed by the first 32 threads. This data will be read into shared memory. To achieve high throughput from/to device memory, we use coalesced memory accesses in which all data accessed by one warp (this is the minimum scheduling unit and it contains 32 threads) falls in the same device memory cache line of size 128 bytes. In Figure 5, six threads fetch the first row from the gray area of block *B*. Then each thread uses the value just fetched to add with the first column in the gray area of block A (which is already read into shared memory). In other words, thread *i *will add *B*[*0*][*i*] with *A*[*j*][*0*] (0 ≤ *j *< 16) and compare this value with *register*[*j*] of thread *i *and update *register*[*j*] if necessary. Then *B*[1][*i*] is added with *A*[*j*] [1] and the result is compared with *register*[*j*]; the register is updated as needed, 0 ≤ *j *< 16. Since threads in the same warp will read data in the same row of block *B*, this reading is coalesced and serviced at the throughput of L1 or L2 cache in case of a cache hit, or at the throughput of device memory, otherwise. Besides, all threads in the same warp use the same value from block *A*, which resides in shared memory. This value can be broadcast to all threads in the same warp.

We note that [11] also employs a *maxKernel *but their kernel is different from ours.

## Results

We benchmarked our algorithms using a PC with a hyperthreaded 6-core Intel i7x980 3.33GHz CPU and 12GB DDR3 RAM. The PC also had NVIDIA Tesla C2050 GPU. Since only two threads may be scheduled per i7 core at any time, a maximum of 12 threads may be gainfully used. We used randomly generated RNA sequences in our experiments. Since the run time of our codes is relatively insensitive to the actual RNA sequence in use due to the fact that the entire computation is to fill out an *n *× *n *matrix, our use of random sequences does not materially impact our conclusions.

### Single and multicore algorithms

In both the codes *OMP*1 and *OMP*2, the work assigned to the threads is well balanced by OpenMP and so best performance is expected using either 6 or 12 threads. Our experiments confirmed this expectation with the use of 6 threads generally being better than the use of 12 threads. So, for our application the overhead of context switching between the two threads assigned to a core when a total of 6 threads are used generally exceeded the gains obtainable from having a second thread ready in case the first thread stalls from memory delay. Table 1 gives the run times for our algorithms *CPU*1, *CPU*2, *OMP*1, and *OMP*2 for *n *values ranging from 3000 to 16000. The columns labeled ratio give the ratios *CPU*1/*OMP*1 and *CPU*2/*OMP*2, respectively. Although we have 6 cores on our CPU, we are able to achieve speedups of almost 5 from the multicore versions. By comparison, the far more complex multicore code of [6], which uses a blocking strategy similar to that used by our GPU code, achieves a simulated speedup of 6.3 with 4 threads. The speedup reported in [6] is referred to as "simulated speedup" because it comes from the use of a multicore simulator rather than from actual speedup measurements on a real muticore computer. However, this simulated speedup ignores several factors such as synchronization overhead that will reduce speedup in a real environment. Further, the simulated speedup of 6.3 is relative to the equivalent of the code *CPU*1. The speedup achieved by *OMP*2 relative to *CPU*1 is between 7.5 and 14.0! We note also that the speedup obtained solely from the use of the caching strategy (i.e., the ratio *CPU*1/*CPU*2) ranges from 1.6 to 3.0.

**Table 1:** Running time (seconds) of different CPU algorithms

*n*	*CPU*1	*OMP*1	*Ratio*	*CPU*2	*OMP*2	*Ratio*
3000	35.9	7.1	5.1	22.3	4.8	4.6
4000	98.1	18.6	5.3	52.8	11.3	4.7
5000	208.1	41.6	5.0	102.9	22.2	4.6
6000	363.7	72.2	5.0	177.5	45.3	3.9
7000	646.1	125.2	5.2	281.3	61.0	4.6
8000	924.4	197.8	4.7	419.6	92.5	4.5
9000	1461.5	291.0	5.0	596.6	129.9	4.6
10000	1927.7	395.0	4.9	819.1	176.9	4.6
11000	2800.8	559.2	5.0	1088.4	234.5	4.6
12000	3525.2	741.4	4.8	1413.6	303.3	4.7
13000	4852.3	978.8	5.0	1795.4	388.4	4.6
14000	6008.9	1250.2	4.8	2243.5	485.2	4.6
15000	7930.0	1641.4	4.8	2757.3	594.0	4.6
16000	10120.0	2380.8	4.3	3343.5	725.4	4.6

### GPU algorithms

**Figure 6:**
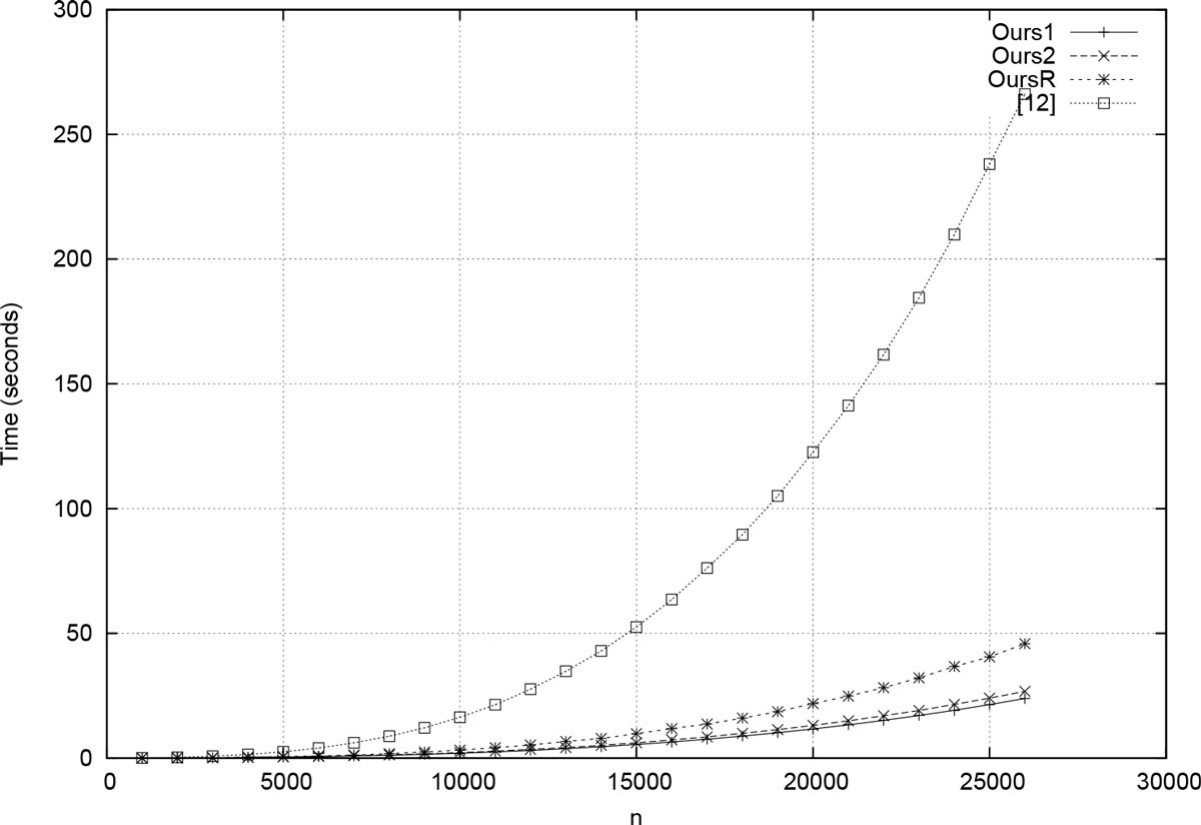
**Plot of running time of GPU algorithms**.

We experimented with three versions of our GPU algorithm. The first is called *Ours1*, which is as described in *Our GPU algorithm *section. In the second version, which is called *Ours2*, device memory usage is reduced by half by storing only the upper triangle of the output matrix. This upper triangle is mapped into a onedimensional array using the standard row-major mapping. Since this version uses only half the device memory used by the other versions, it may be used on larger instances. In the third version, which is called *OursR*, we replaced our *maxKernel *with the kernel described in [11]. Since we were unable to get the GPU code of [11], the kernel used by us was actually one we wrote based on the description provided in [11]. These three codes were benchmarked against each other as well as against the GPU Nussinov code of [12]. The maximum size of sequence *Ours*2 can handle is 37000 while the other versions can handle sequences of size up to 26000. *Ours*2 runs slightly slower than *Ours*1 as shown in Table 2. So, *Ours*2 is recommended only when the instance size is large enough to make *Ours*1 nonfeasible. Table 2 and Figure 6 show the running time for the four different GPU codes. *Ratio*1 in Table 2 shows the speedup of *Ours*1 relative to [12] ([12]/*Ours*1). *Ratio*2 shows *OursR*/*Ours*1. As can be seen, *Ours*1 is up to 1.9 times as fast as *OursR *indicating that a corresponding speedup could be obtained for Zuker's algorithm by replacing the maximum finding kernel used in [11] with our kernel for this operation. Also, *Ours*1 is between 3.0 and 11.1 times as fast as the GPU algorithm of [12].

**Table 2:** Running time (seconds) of different GPU algorithms

*n*	*Ours*1	*Ours*2	[12]	*OursR*	*Ratio*1	*Ratio*2
2000	0.1	0.1	0.3	0.1	3.0	1.0
6000	0.6	0.7	4.0	0.8	6.7	1.3
10000	1.9	2.2	16.4	3.2	8.6	1.7
14000	4.5	5.1	43.0	7.9	9.6	1.8
18000	8.8	9.9	89.5	16.0	10.2	1.8
22000	15.1	16.9	161.7	28.2	10.7	1.9
26000	23.9	26.7	266.3	45.8	11.1	1.9
37000	-	71.5	-	-	-	-

### Single core vs multicore vs GPU

Table 3 gives the speedup obtained by *Ours*1 relative to *CPU*2 and *OMP*2. Using a GPU, we can do the Nussinov computations up to 522.6 times as fast as using a cache efficient single core code and up to 113.4 times as fast as using a 6-core cache efficient code! Compared to the naive single-core code CP U 1, our GPU codes provides a speedup of up to 1582!

**Table 3:** Speedup of Ours1 relative to other versions

*n*	*CPU*2	*OMP*2	*n*	*CPU*2	*OMP*2
3000	157.0	33.8	10000	424.4	91.7
4000	224.7	48.1	11000	441.4	95.1
5000	259.8	56.1	12000	465.9	100.0
6000	302.9	77.3	13000	472.3	102.2
7000	341.8	74.1	14000	496.9	107.5
8000	376.0	82.9	15000	503.3	108.4
9000	392.2	85.4	16000	522.6	113.4

## Conclusions

We have developed simple and efficient single and multi-core algorithms as well as an efficient GPU code for RNA folding based on Nussinov's equations [2]. Our cache efficient single-core algorithm provides a speedup between 1.6 and 3.0 relative to a naive straightforward single core code. The multicore version of the cache efficient single core algorithm provides a speedup, relative to the naive single core algorithm, between 7.5 and 14.0 on a 6 core hyperthreaded CPU. Our GPU algorithm, *Ours*1, for the NVIDIA C2050 is up to 1582 times as fast as the naive single core algorithm and between 3.0 and 11.1 times as fast as the fastest previously known GPU algorithm for Nussinov RNA folding. With the available 3GB device memory on an NVIDIA GPU, *Ours*1 is able to handle sequences of length up to 26000. Sequences of length between 26000 and 37000 may be handled using *Ours*2, which uses a onedimensional array mapping of the upper triangle of the output matrix rather than a two-dimensional array that represents the full output matrix. *Ours*2, however, runs slightly slower than *Ours*1. Our methods can be used to speedup up RNA folding using Zuker's equations as well [3,11].

## List of abbreviations used

RNA: RiboNucleic Acid; GPU: Graphics Processing Unit; PCI-Express: Peripheral Component Interconnect Express; CUDA: Compute Unified Device Architecture; GCUPS: Billion Cell Updates per Second; SM: Streaming Multiprocessors; DRAM: Dynamic Random-Access Memory; TFLOPS: Trillion Floating Point Operations Per Second; GFLOPS: Billion Floating Point Operations Per Second; I/O: Input/Output; CPU: Central Processing Unit.

## Competing interests

The authors declare that they have no competing interests.

## Authors' contributions

JL, SR, and SS developed the GPU algorithms, and analyzed the experimental results, and wrote the manuscript. JL also programmed and debugged the GPU algorithms and ran the experiments.

## Declarations

Publication of this supplement was funded, in part, by the National Science Foundation under grants CNS0963812, CNS1115184, CNS0905308, and the National Institutes of Health under grant R01-LM010101.

This article has been published as part of *BMC Bioinformatics *Volume 15 Supplement 8, 2014: Selected articles from the Third IEEE International Conference on Computational Advances in Bio and Medical Sciences (ICCABS 2013): Bioinformatics. The full contents of the supplement are available online at http://www.biomedcentral.com/bmcbioinformatics/supplements/15/S8.
